# Photoinduced Antibacterial Activity of the Essential Oils from *Eugenia brasiliensis* Lam and *Piper mosenii* C. DC. by Blue Led Light

**DOI:** 10.3390/antibiotics8040242

**Published:** 2019-11-28

**Authors:** Rakel Olinda Macedo da Silva, José Walber Gonçalves Castro, Orlando de Menezes Dantas Junior, Ana Carolina Justino de Araújo, Maria Karollyna do Nascimento Silva Leandro, Raíra Justino Oliveira Costa, Luciely Leite Pinto, Lívia Maria Garcia Leandro, Luiz E. da Silva, Wanderlei do Amaral, Lucas D. Parabocz, Aurea P. Ferriani, Bruna Garcia, Beatriz H. L. N. Sales Maia, Janaína Esmeraldo Rocha, Camila Fonseca Bezerra, Thiago Sampaio de Freitas, Maria Socorro Costa, Fábia Ferreira Campina, Edinardo Fagner Ferreira Matias, Marcello Iriti, Henrique Douglas Melo Coutinho

**Affiliations:** 1 Department of Biomedicine, University Center Dr. Leão Sampaio, Juazeiro do Norte 63040-005, Brazil; rakelolinda@leaosampaio.edu.br (R.O.M.d.S.); walbercastro1@hotmail.com (J.W.G.C.); orlandodm_fs@hotmail.com (O.d.M.D.J.); karollynasilva@leaosampaio.edu.br (M.K.d.N.S.L.); rairajustino@hotmail.com (R.J.O.C.); lucielyleite@gmail.com (L.L.P.); livialeandro@leaosampaio.edu.br (L.M.G.L.); edinardomatias@gmail.com (E.F.F.M.); 2 Department of Biological Chemistry, Regional University of Cariri, Crato 63105-000, Brazil; caroljustino@outlook.com (A.C.J.d.A.); janainaesmeraldo@gmail.com (J.E.R.); camilawasidi@gmail.com (C.F.B.); thiagocrato@hotmail.com (T.S.d.F.); corrinha_live@yahoo.com.br (M.S.C.); fabiacampina@gmail.com (F.F.C.); hdmcoutinho@gmail.com (H.D.M.C.); 3 Setor Litoral, Federal University of Paraná, Curitiba 80.060-000, Brazil; luiz_everson@yahoo.de (L.E.d.S.); wdoamaral@hotmail.com (W.d.A.); diovaniparabocz@hotmail.com (L.D.P.); aurea.portes@hotmail.com (A.P.F.); klebisbruna@gmail.com (B.G.); bhsalesmaia@gmail.com (B.H.L.N.S.M.); 4 Department of Agricultural and Environmental Sciences, Milan State University, via G. Celoria 2, 20133 Milan, Italy

**Keywords:** bacterial resistance, blue light, *Escherichia coli*, *Eugenia brasiliensis*, *Piper mosenii*, *Staphylococcus aureus*

## Abstract

The objective of this work was to evaluate the phytochemical composition and the antibacterial and antibiotic-modulating activities of the essential oils of *Eugenia brasiliensis* Lam (OEEb) and *Piper mosenii* C. DC (OEPm) singly or in association with blue LED (Light-emitting diode) light. The antibacterial and antibiotic-modulatory activities of the essential oils on the activity of aminoglycosides were evaluated to determine the minimum inhibitory concentration (MIC, μg/mL) in the presence or absence of exposure to blue LED light. The chemical analysis showed α-pinene and bicyclogermacrene as major constituents of OEPm, whereas α-muurolol was the main compound of OEEb. Both OEEb and OEPm showed MIC ≥ 512 μg/mL against the strains under study. However, the association of these oils with the blue LED light enhanced the action of the aminoglycosides amikacin and gentamicin. In conclusion, the association of aminoglycosides with the blue LED light and essential oils was effective against resistant bacteria.

## 1. Introduction

Bacterial resistance is a phenomenon caused by the evolution or adaptation of microorganisms to antibiotics, through several molecular mechanisms [1]. Infections caused by multi-drug resistant microorganisms represent one of the major public health problems worldwide, both because of increased morbidity and mortality and due to high hospitalization costs [2,3].

The World Health Organization (WHO) published in February 2017 a document indicating which bacteria should be prioritized in studies involving new antibiotics, because they have currently few treatment options. These bacteria include Gram-negative strains of Enterobacteria resistant to carbapenems and cephalosporins, such as *Escherichia coli* and *Klebsiella pneumoniae*, as well as Gram-positive strains, such as Methicillin-resistant *Staphylococcus aureus* (MRSA) and Vancomycin-resistant *S. aureus* (VRSA) [4].

In this context, natural products, such as essential oils, have been widely studied in the treatment of various diseases. Because they present secondary metabolites with important biological activities, these substances have shown promising pharmacological effects [5,6,7]. In addition, several studies have shown that the association of essential oils with antibiotics has a synergistic effect, especially against strains of multi-resistant bacteria [8]. 

*Eugenia brasiliensis* Lam (Myrtaceae) is a native forest species popularly known as “grumixama” [9]. In Brazil, this plant is used as food or for its medicinal properties [10,11]. In fact, previous studies demonstrated that this plant exhibits anti-inflammatory, antimicrobial, antidepressant, and antioxidant activities [12].

On the other hand, *Piper mosenii* C. has no popular name or use as a medicinal plant reported in the literature [13,14]. Nevertheless, many species belonging to this genus have economic importance, besides having medicinal properties, such as antimicrobial and antiproliferative activities [15,16,17].

Light-emitting diode (LED) therapy has been employed in the treatment of some infections [18]. This therapy has presented countless benefits for health, as it promotes wound healing, tissue repair, and other effects on various skin conditions, without causing side effects to patients [19].

Therefore, this study aimed to evaluate the chemical composition and the antibacterial effect of the essential oils obtained from *Eugenia brasiliensis* Lam and *Piper mosenii* C. DC., as well investigate their modulatory potential in association with blue LED light and aminoglycoside antibiotics against standard and multi-resistant strains of *Escherichia coli* and *Staphylococcus aureus*.

## 2. Results

### 2.1. Chemical Profile of the Essential Oils

**Figure 1:**
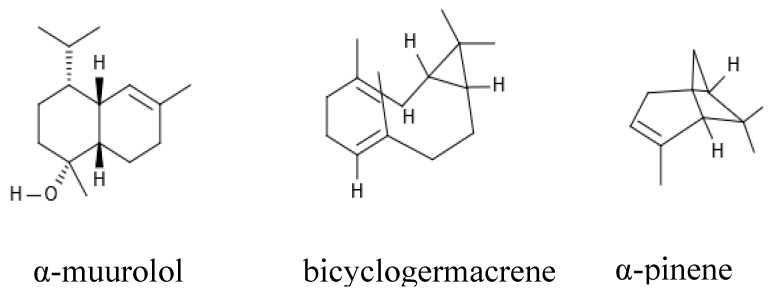
Chemical structure of the main phytocompounds from the essential oils.

After extraction, the essential oils obtained from the leaves of *Eugenia brasiliensis* Lam and *Piper mosenii* C. DC. presented yields of 0.27% and 0.49%, respectively. A chemical analysis of these oils detected the presence of 20 chemical components in OEEb and 18 components in OEPm, as shown in Table 1 and Table 2. Additionally, this analysis showed α-pinene and bicyclogermacrene as major constituents of OEPm, whereas α-muurolol was the main compound identified in OEEb (Figure 1).

The composition of the essential oil of leaves of *E. brasiliensis* Lam was analyzed by previous studies, which demonstrated the presence of several chemical components, such as spatulenol, α- and β-pinene, τ-cadinol, and α- and β-selinene [20,21]. A study by Moreno et al. [22], described the terpene 1,8-cineol as a major constituent present in the essential oil of this plant. However, this compound was found in lower quantity in the present study, indicating that the composition of the essential oil may vary according to the conditions of the sample used in each study. On the other hand, in the study by Fidyt et al. [23], α-β-carrageenan, α-copaene, and pinene (α and β) were identified as major constituents, corroborating with the present study which described α-pinene as a major component of the essential oil.

Bernuci et al. [16] described for the first time the composition of the essential oil of the species *Piper mosenii*, mentioning the presence of compounds such as β-pinene, *p*-cymene, α-thujene, (*E*)-cariophyllene, bicyclogermacrene, γ-cadinene, *trans*-calamene, *allo*-aromadendrene, globulol, viridiflorol, and α-cadinol. It is worth noting that there are far fewer studies on this species than on *E. brasiliensis*.

Differences in the composition and concentration of components of essential oils in studies with the same plant may be justified by variations on ecological and environment factors such as climate, relief, temperature, and soil type, as well as the part of the plant used in each case [24]. According to Radünz et al. [25], the accumulation of active principles can also vary according to the period of the year or due to genetic and physiological factors of the species, besides the age and the drying process of the leaves.

### 2.2. Antibacterial Activity by Direct Contact 

An evaluation of the antibacterial activity of the essential oils of the two species demonstrated that OEPm was active against *Staphylococcus aureus*, with a MIC of 512 μg/mL (Table 3). Exposure to blue LED light did not modify the MICs of the treatments, indicating that, under the conditions of the present study, the blue light exhibited no modulating effect on the antibacterial activity of the essential oil obtained from *Piper mosenii*.

On the other hand, the essential oil of *Eugenia brasiliensis* Lam did not present clinically relevant antibacterial activity against strains of *S. aureus* and *E. coli* with MIC values ≥ 1024 µg/mL. These data differ from those of a study by Magina et al. [26], in which an essential oil obtained from the leaves of this species presented moderate activity against *E. coli* (MIC = 624.9 μg/mL) and strong activity against *S. aureus* (MIC = 156.2 μg/mL) in tests performed by the microdilution method. These differences may be related to differences in the chemical composition of the products used in each study, because, in the essential oil obtained in the work of those authors, spatulenol (12.6%) and s-cadinol (8.7%) were the major constituents, whereas the essential oil obtained in the present study had α-muurolol (12.01%) as principal constituent.

Regarding the antibacterial activity of *Piper mosenii* C.DC., it is emphasized that both standard and multi-resistant strains of *Staphylococcus aureus* had their growth inhibited by the essential oil of this plant. These data are supported by a study using the diffusion disc method that showed that the *Piper* species have antibacterial potential. It was demonstrated that the crude ethanolic extract, in addition to the hexane and chloroform fractions of the *Piper mollicomum* leaves, showed antibacterial activity against standard strains of *S. aureus* [27]. A study demonstrated the bioactivity of α-pinene, a major constituent of the essential oil of *P. mosenii* C. DC., against strains of *Staphylococcus aureus*, *Salmonella pullorum*, and *Klebsiella pneumoniae* as well as other bacterial species [28]. Moreover, this compound exhibited significant activity against *Mycobacterium tuberculosis* strains [29] and its positive enantiomer demonstrated to be active against Methicillin-resistant *Staphylococcus aureus* (MRSA), *Candida albicans*, and *Cryptococcus neoformans*, demonstrating an antimicrobial effect that appears to be related to the chemical structure [30]. 

It is hypothesized that bicyclogermacrene (the second major constituent of the oil) contributed to the antibacterial effect of *P. mosenii* C. DC. However, the isolated action of this against both standard and multi-resistant bacterial strains remains to be investigated. 

Interestingly, the essential oils of *P. mosenii* and *E. brasiliensis* showed no activity against *E. coli*. As Gram-negative bacteria, *E. coli* has a cell wall rich in lipopolysaccharides that inhibit the entry of several antimicrobial substances. Therefore, these microorganisms are frequently less sensitive to the action of essential oils [31].

### 2.3. Modulating Effect of Essential Oils in Association with Blue LED Light on the Activity of Aminoglycosides

**Figure 2:**
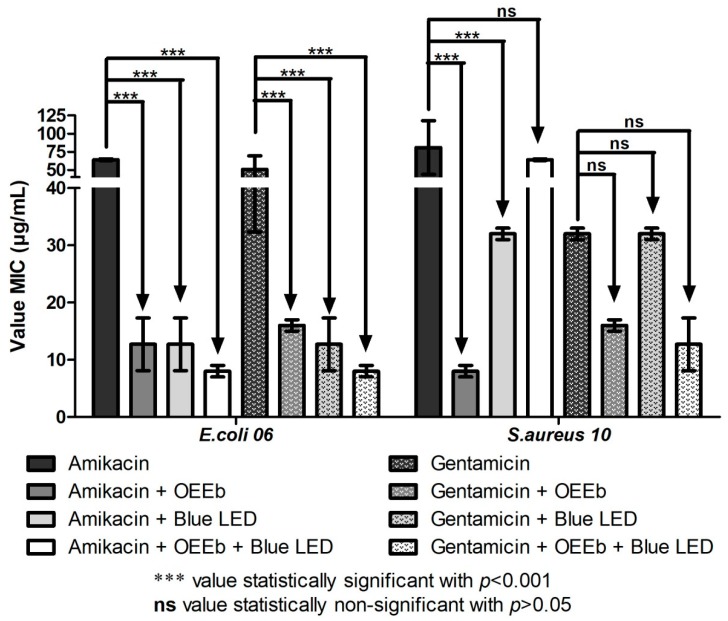
Geometric mean of the minimum inhibitory concentration (μg/mL) of the aminoglycosides alone or in association with the OEEb and the blue LED light against the MDR strains *E. coli* 06 and *S. aureus* 10.

The antibacterial effect of the blue LED light is well known in the literature [18,19]. However, against both bacterial strains assayed, the blue LED light alone demonstrated a MIC ≤ 1024 μg/mL. In the modulation tests, association of OEEb with amikacin and gentamicin presented a synergistic antibacterial effect against the multi-resistant strain of *Escherichia coli* (Figure 2). The effect of this association was further enhanced was by exposure to blue LED light. In the tests with the multi-resistant strain of *Staphylococcus aureus*, association with OEEb or blue LED light increased the activity of amikacin. However, the simultaneous association of OEEb with blue LED light and the antibiotics did not present a significant antibacterial effect. 

**Figure 3:**
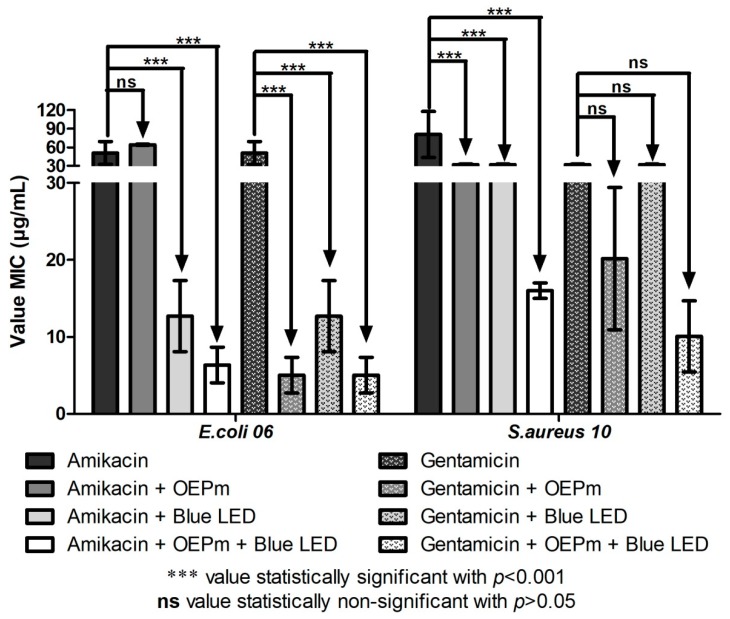
Geometric mean of the minimum inhibitory concentration (μg/mL) of the aminoglycosides alone or in association with the OEPm and the blue LED light against the MDR strains *E. coli* 06 and *S. aureus* 10.

In the test with OEPm, association of this substance with amikacin did not affect the activity of this antibiotic against *E. coli* (Figure 3). However, when this combination was exposed to blue LED light, the antibacterial effect was potentiated, indicating that light exposure stimulated synergistic interactions between the treatments. Interestingly, in the tests with gentamicin, blue light, and OEPm, all combinations showed synergism against this microorganism. Considering these combinations against *S. aureus*, all conditions presented synergism with amikacin, but no modulation was observed with gentamicin.

These results indicate that blue LED light act as a modulator of the antibiotic activity of aminoglycosides, especially in the presence of the essential oils obtained from the plants under study. It is worth noting that the modulating effect of blue light associated with essential oils was stronger against *E. coli*, which resisted the modulating effect of OEPm in the absence of light exposure.

The results obtained in the present study corroborate those described by Pereira et al. [32]. Through the gaseous contact method, the authors observed a synergistic effect from the association of blue LED light with the essential oil of *Eugenia jambolana* and antibiotics against strains of *Escherichia coli* and *Staphylococcus aureus*. According to the authors, investigation of the combined effect of essential oils and LED lights may represent a key step in development of novel therapies against infections caused by resistant bacteria.

Promising results were also obtained by Matias et al. [33] studying a combination of blue LED light, antibiotics, and the essential oil of *Cordia verbenacea* DC. by the gaseous contact method. In the presence of ciprofloxacin and norfloxacin, the association of the oil and blue light presented a synergistic effect against *E. coli* and *S. aureus*. However, in the presence of gentamicin and amikacin, the same combination was effective only against *S. aureus*. These results differ partially of those obtained in the present study, which may have occurred because of differences on the methodologies or in the chemical constitution of the essential oils analyzed.

According to Caffarel-Salvador et al. [34], the use of photodynamic therapy is a new form of antimicrobial therapy, which is already used for treatment of cancerous skin lesions and has been efficiently evaluated in the treatment of infections. This is a low-cost method that provides a biophysical and biochemical rebalancing in the treatment of diseases, besides being non-invasive [35].

In terms of mechanism, phototherapy can cause activation of chemical compounds, as previously reported in a study by Brito et al. [36]. This study demonstrated that inactive compounds present in the skin of the amphibian *Rhinella jimi* acquired an antimicrobial activity after association with ultraviolet light.

The blue LED light can act by eliminating both Gram-positive and Gram-negative bacteria by inducing oxidative stress [32]. This effect is triggered when the light is associated with chemical compounds found in medicinal plants that act as photosensitizers. Thus, the compounds are excited, which results in the formation of intracellular reactive oxygen species (ROS), causing oxidation of essential constituents of the bacterial cells with consequent death of these microorganisms [37,38].

Therefore, it is suggested that components of the essential oils used in the present study may act as photosensitizing substances, because they had the antibacterial effect improved in the presence of blue LED light, modulating the activity of aminoglycosides and consequently reducing the MIC of these antibiotics. This phenomenon has great clinical relevance, because this type of therapy may reduce the dose of antibiotics required to treat infections, minimizing their side effects [39]. Furthermore, association of LED light and essential oils can be used to improve the effectiveness of conventional antibiotics against resistant bacteria.

## 3. Methodology

### 3.1. Plant Material and Extraction of Essential Oils

The fresh leaves of *Eugenia brasiliensis* Lam were collected in December 2015 in Atalanta, Santa Catarina, Brazil with the coordinates 25°29.830′ S and 49°00.919′ W at an altitude of 640 m. A voucher specimen was deposited in the Dr. Roberto Miguel Klein herbarium of the Regional University of Blumenau under the number FURB32094. The fresh leaves of *Piper mosenii* C. DC. were collected in August 2015 in the Bom Jesus Biological Reserve in the municipality of Guaraqueçaba, Paraná, Brazil, with coordinates 25°13.939′ S and 48°34.821′ W and an altitude of 13 m. A voucher specimen was deposited in the Herbarium of the Municipal Botanical Museum (MBM) in Curitiba under the number MBM396409 (SISGEN No. A37FBDC).

The extraction of the essential oils from *Eugenia brasiliensis* Lam (OEEb) and *Piper mosenii* C. DC (OEPm) was performed by the hydrodistillation method using the Clevenger type apparatus. Fresh leaves of *E. brasiliensis* (100 g) and dried leaves of *P. mosenii* (50 g) were crushed, placed in a glass flask with 1.0 L of distilled water, and boiled for 2.5 h for extraction of OEEb and 4.5 h for extraction of OEPm [40]. The leaves were dried with an electric dryer (FANEM, Mod. 320 SE, FANEM ltda., Campinas, Brazil) with air circulation at 40 °C for 24 h. After extraction, the samples were collected with a precision pipette and conditioned in a freezer until the analysis. To determine the essential oil content in dry basis, the total mass of each essential oil obtained was considered in relation to the amount of the dry mass of the botanical material used in the extraction.

### 3.2. Determination of the Chemical Composition of the Essential Oils

The chemical constituents of the essential oils were identified by gas chromatography coupled to mass spectrometry (GC/MS). The essential oils were diluted to a concentration of 1% in dichloromethane and 1.0 μL of the solution was injected with a 1:20 flow split into a chromatograph (Agilent 6890, Palo Alto, CA, USA) coupled to a mass selective detector (Agilent 5973N, Palo Alto, CA, USA). The injector was maintained at 250 °C and the constituents were isolated in a HP-5MS capillary column (5% phenyl–95% dimethylpolysiloxane, 30 mm × 0.25 mm × 0.25 μM) using helium as carrier gas (1.0 mL min^−1^). The oven temperature was programmed from 60 to 240 °C at a rate of 3 °C min^−1^. The mass detector was operated in electronic ionization mode (70 eV), at a rate of 3.15 sweeps s^−1^ and a mass band of 40–450 μm. The transfer line was maintained at 260 °C, with ion source at 230 °C and analyzer (quadrupole) at 150 °C. 

For quantification, the diluted samples were injected into a chromatograph (Agilent 7890A, Palo Alto, CA, USA) equipped with a flame ionization detector (FID), operated at 280 °C. The same column and analytical conditions described above were employed, except for the carrier gas, which was hydrogen, at a flow rate of 1.5 mL min^−1^. The percentage composition was obtained by the electronic integration of the FID signal by dividing the area of each component by the total area (area%).

The identification of the chemical constituents was performed by comparing their mass spectra with those of spectral libraries Wiley (1994) and NIST (2016) and by their linear retention indexes, calculated from the injection of a homologous series of hydrocarbons (C7–C26) and compared with data from the literature [41].

### 3.3. Antibiotics, Culture Media and Microorganisms

The liquid antibiotics gentamicin and amikacin were obtained from LaborClin, Belo Horizonte, Brazil. Heart Infusion Agar (HIA) and Brain Heart Infusion (BHI) culture media were acquired from HIMEDIA.

The microorganisms used in the tests were provided by the Laboratory of Microbiology and Molecular Biology (LMBM) of the Regional University of Cariri (URCA). The microorganisms used in the tests were provided by the Laboratory of Microbiology and Molecular Biology (LMBM) of the Regional University of Cariri (URCA). The standard strains were characterized and classified by the American Type Culture Collection (ATCC): *Escherichia coli* (EC ATCC 25922) and *Staphylococcus aureus* (SA ATCC 25923). The clinical isolates *Escherichia coli* (EC 06) and *Staphylococcus aureus* (SA 10) were characterized and classified by laboratories of clinical analysis from the region, and the resistance profile was determined by disk diffusion method. The resistance profile is available in the literature [32,33].

### 3.4. Preparation of Test Solutions

The test solutions were prepared using 10 mg of each oil diluted in 0.5 mL of dimethyl sulfoxide (DMSO). Each solution was placed in a falcon tube and 9265 mL distilled water was added, resulting in a final concentration of 1024 μg/mL. The solutions of the oils at this concentration were used in the antibacterial and modulation tests. The antibiotics used in the tests were also prepared at an initial concentration of 1024 μg/mL.

### 3.5. Determination of the Minimum Inhibitory Concentration (MIC) by Direct Contact

Bacterial samples were seeded in Petri dishes containing HIA and placed in an oven at 37 °C for 24 h to grow. After this period, the bacterial samples were diluted in test tubes. Then, the turbidity of the solution was determined according to the 0.5 of the McFarland scale (10^8^ UFC/mL) [42].

To evaluate the antibacterial activity, 100 μL of the inoculum solution were added to each well of the microdilution plate. Then, the treatments were performed using 100 μL of each oil per column at final concentrations ranging from 1024 to 0.5 μg/mL. Of note, all treatments were performed in triplicate. The plates were taken to an oven at 37 °C for 24 h. Then, each well was added with 20 μL of resazurin (a colorimetric indicator) and the 1 h later the MIC was determined by ocular observation [42].

The antibiotic activity modulation assay was performed using amikacin and gentamicin according to the method described by Coutinho et al. [43]. Briefly, test tubes were added with 150 μL of the bacterial suspension in a solution containing 10% BHI medium and the essential oils at subinhibitory concentrations (MIC/8). Control tubes were prepared using 150 μL of the bacterial suspension in a solution containing 10% BHI medium. Then, 100 μL of these solutions were transferred to corresponding wells in the plate and 100 μL of the antibiotic were added to the first well and serially diluted, with the final concentrations ranging from 1024 to 0.5 μg/mL. The antibiotic alone was used as a positive control. All the assays were performed in triplicate and the MICs were determined as described above.

### 3.6. Evaluation of Modulating Activity Associated with Blue LED Light Exposure

This protocol was carried out using the Light Emitting Diodes (LED) device (brand NEW Estética®). In the experiments, the blue light was used with a wavelength of 415 nm pre-determined by the apparatus, with 5 cm of distance between the LED and the biological material.

The experimental protocol followed initially the same methodology used to evaluate the antibacterial and modulating activities by direct contact. Thereafter, the plates were exposed to blue LED light for 20 min, with a total dose of 40 mW/cm^2^ (180 lumens), and subsequently incubated at 37 °C for 24 h. The assays were performed in triplicate and sodium resazurin was used to determine the MIC.

### 3.7. Statistical Analysis

The microbiological results were expressed as geometric mean and analysis of results was applied to two-way ANOVA followed by Bonferroni posttests using GraphPad Prism 6.0 software. Results with *p* < 0.05 were considered as statistically significant.

## 4. Conclusions

The results obtained in the present study demonstrate that, when associated with aminoglycosides, the essential oils of *Eugenia brasiliensis* Lam and *Piper mosenii* C. DC. presented antibiotic-modulating activities, especially against the Gram-negative strain. This effect was potentiated by exposure to blue LED light, possibly through a photosensitizing mechanism.

In conclusion, the association of LED light and essential oils can be used to improve the effectiveness of conventional antibiotics against resistant bacteria, although further research is needed to determine the impact of such combined therapy in medicine.

## Figures and Tables

**Table 1:** Phytocompounds identified by GC/MS analysis from the essential oil of *Eugenia brasiliensis* Lam (OEEb).

RI Calculated	Composition	%
935	tricyclene	7.27
980	sabinene	5.39
1030	limonene	8.96
1034	1,8-cineole	4.84
1103	Linalool	1.54
1196	α-terpineol	1.53
1425	(E)-caryophyllene	2.69
1503	β-macrocarpene	2.30
1521	γ-cadinene	1.22
1530	δ-cadinene	5.26
1576	longipinanol	1.01
1587	spathulenol	6.16
1593	thujopsan-2-α-ol	6.11
1603	cubeban-11-ol	4.40
1613	5-*epi*-7-*epi*-α-eudesmol	2.12
1624	1,10-di-*epi*-cubenol	1.23
1638	1-*epi*-cubenol	4.35
1651	α-muurolol (=torreyol)	12.01
1657	valerianol	3.60
1665	selin-11-em-4-α-ol	7.10

**Table 2:** Phytocompounds identified by GC/MS analysis from the essential oil of *Piper mosenii* C. DC. (OEPm).

RI Calculated	Composition	%
935	α-pinene	14.59
980	β-pinene	2.72
1445	aromadendrene	2.12
1460	α-humulene	3.85
1468	*allo* aromadendrene	3.74
1483	germacrene D	1.08
1487	γ-muurolene	2.77
1493	β-selinene	1.92
1504	bicyclogermacrene	12.25
1531	δ-cadinene	4.74
1587	fokienol	8.43
1592	globulol	6.15
1613	sesquithuriferol	1.5
1619	*epi*-cedrol	1.89
1652	α-cadinol	2.02
1665	*neo*-intermedeol	1.62
1670	intermedeol	1.66
1695	caryophyllene acetate	4.73

**Table 3:** Geometric mean of the minimum inhibitory concentration (μg/mL) of the essential oils from *Eugenia brasiliensis* Lam (OEEb) and *Piper mosenii* C. DC. (OEPm), alone and in association with the ble LED light. *Staphylococcus aureus* (SA); *Escherichia coli* (EC).

Bacterial Strains	OEEb		EOPm	
	**Normal light**	**Blue LED Light**	**Normal light**	**Blue LED Light**
**SA ATCC 25923**	≥1024	≥1024	512	512
**SA 10**	≥1024	≥1024	512	512
**EC ATCC 25922**	≥1024	≥1024	≥1024	≥1024
**EC 06**	≥1024	≥1024	≥1024	≥1024

## References

[1] Sampaio P.S., Sancho L.G., Lago R.F. (2018). Implementação da nova regulamentação para prescrição e dispensação de antimicrobianos: Possibilidades e desafios. Cad. Saúde Colet..

[2] Nathan C., Cars O. (2014). Antibiotic resistance-problems, progress, and prospects. N. Engl. J. Med..

[3] Piltcher O.B., Kosugi E.M., Sakano E., Mion O., Testa J.R.G., Romano F.R., Santos M.C.J., Di Francesco R.C., Mitre E.I., Bezerra T.F.P. (2018). How to avoid the inappropriate use of antibiotics in upper respiratory tract infections? A position statement from an expert panel. Braz. J. Otorhinolaryngol..

[4] Shrivastava S.R., Shrivastava P.S., Ramasamy J. (2018). World health organization releases global priority list of antibiotic-resistant bacteria to guide research, discovery, and development of new antibiotics. J. Med. Soc..

[5] Hosseinzadeh S.B., Boerger M.R.T., Negrelle R.B., Bergo C. (2017). Effect of ultrasound and infrared drying methods on quantitative and qualitative characteristics of *Satureja bachtiarica* essential oil. J. Essent. Oil-Bear. Plants.

[6] Souza M.R., Maingredy X., Milton B., Dryelle S.P., Júnior M. (2016). Fioterápicos no tratamento de transtornos de ansiedade. Eletron. J. Pharm..

[7] Sobrinho T.J.S.P., Castro V.T.N.A., Saraiva A.M., Almeida D.M., Tavares E.A., Amorim E.L.C. (2011). Phenolic content and antioxidant capacity of our *Cnidoscolus* species (Euphorbiaceae) used as ethnopharmacologicals in Caatinga. Afr. J. Pharm. Pharmacol..

[8] Coutinho H.D.M., Costa J.G.M., Siqueira-Júnior J.P., Falcão-Silva V., Lima E. (2012). Fruits to potentiate the antibiotic activity: The effect of *Eugenia uniflora* and *Eugenia jambolanum* L. against MRSA. Acta Aliment..

[9] Lorenzi H. (2002). Árvores Brasileiras: Manual de identificação e cultivo de plantas arbóreas nativas do Brasil.

[10] Benfatti C.S., Cordova S.M.D., Guedes A., Magina M.D.A., Cordova C.M.M.D. (2010). Atividade antibacteriana in vitro de extratos brutos de espécies de *Eugenia* sp frente a cepas de molicutes. Rev. Pan-Amaz. Saúde..

[11] Suguino E., Martins A.N., Minami K., Narita N., Perdoná M.J. (2011). Efeito da porosidade do substrato casca de *Pínus* no desenvolvimento de mudas de Grumixameira. Rev. Bras. Frut. Espec..

[12] Magina M.D.A., Dalmarco E.M., Dalmarco J.B., Colla G., Pizzolatti M.G., Brighente I.M.C. (2012). Bioactive triterpenes and phenolics of leaves of *Eugenia brasiliensis*. Quim. Nova.

[13] BFG-The Brazil Flora Group (2015). Growing knowledge: An overview of seed plant diversity in Brazil. Rodriguésia.

[14] Christ J.A., Hollunder R.K., Carvalho M.S., Ferreira M.F.D.S., Garbin M.L., Carrijo T.T. (2018). DNA fingerprinting based on SSR amplification profiles for *Piper* species identification (Piperaceae). Acta Bot. Bras..

[15] Gogosz A.M., Boerger M.R.T., Negrelle R.B., Bergo C. (2012). Anatomia foliar comparativa de nove espécies do gênero *Piper* (Piperaceae). Rodriguésia.

[16] Bernuci K., Iwanaga C.C., Fernandez-Andrade C.M.M., Lorenzetti F.B., Torres-Santos E.C., Faiões V.D.S., Gonçalves J.E., Amaral W., Deschamps C., Scodro R.B.L. (2016). Evaluation of chemical composition and antileishmanial and antituberculosis activities of essential oils of *Piper* species. Molecules.

[17] Velandia S.A., Quintero E., Stashenko E.E., Ocazionez R.E. (2018). Actividad antiproliferativa de aceites esenciales de plantas cultivadas en Colombia. Acta Biol. Colomb..

[18] Lipovsky A., Nitzan Y., Gedanken A., Lubart R. (2010). Visible light-induced killing of bacteria as a function of wavelength: Implication for wound healing. Lasers Surg. Med..

[19] Dourado K.B.V., Carnevali-Junior L.C., Paulo R.J.F., Gomes A.C. (2011). LEDTERAPIA, uma nova perspectiva terapêutica ao tratamento de doenças da pele, cicatrização de feridas e reparação tecidual. Ensaios e Ciência: Ciências agrárias, biológicas e da saúde.

[20] Apel M.A., Sobral M., Schapoval E.E.S., Henriques A.T., Menut C., Bessiere J.M. (2004). Essential oils from *Eugenia* species. Part VII: Sections Phyllocalyx and Stenocalyx. J. Essent. Oil Res..

[21] Fischer D.C.H., Limberger R.P., Henriques A.T., Moreno P.R. (2005). Essential oils from leaves of two *Eugenia brasiliensis* specimens from Southeastern Brazil. J. Essent. Oil Res..

[22] Moreno P.R.H., Lima M.E.L., Sobral M., Young M.C.M., Cordeiro I., Apel M.A., Limberger R.P., Henriques A.T. (2007). Essential oil composition of fruit colour varieties of *Eugenia brasiliensis* Lam. Sci. Agric..

[23] Fidyt K., Fiedorowicz A., Strządała L., Szumny A. (2016). *β*-Caryophyllene and *β*-caryophyllene oxide-natural compounds of anticancer and analgesic properties. Cancer Med..

[24] Andrade M.A., Cardoso M.G., Batista L.R., Mallet A.C.T., Machado S.M.F. (2012). Óleos essenciais de *Cymbopogon nardus*, *Cinnamomum zeylanicum* e *Zingiber officinale*: Composição, atividades antioxidante e antibacteriana. Rev. Ciên. Agron..

[25] Radünz L.L., Melo E.C., Barbosa L.C.A., Rocha R.P., Berbert P.A. (2012). Rendimento extrativo de cumarina de folhas de guaco (*Mikania glomerata* Sprengel) submetidas a diferentes temperaturas de secagem. Rev. Bras. Plantas Med..

[26] Magina M.D.A., Dalmarco E.M., Wisniewski Jr A., Simionatto E.L., Dalmarco J.B., Pizzolatti M.G., Brighente I.M.C. (2009). Chemical composition and antibacterial activity of essential oils of *Eugenia* species. J. Nat. Med..

[27] Alves H.S., Rocha W.R.V., Fernandes A.F.C., Nunes L.E., Pinto D.S., Costa J.I.V., Chaves M.C.O., Catão R.M.R. (2016). Actividad antimicrobiana de productos obtenidos a partir de especies de *Piper* (Piperaceae). Rev. Cuba. Plant. Med..

[28] Dorman H.J.D., Deans S.G. (2000). Antimicrobial agents from plants: Antibacterial activity of plant volatile oils. J. Appl. Microbiol..

[29] Sieniawska E., Sawicki R., Swatko-Ossor M., Napiorkowska A., Przekora A., Ginalska G., Augustynowicz-Kopec E. (2018). The effect of combining natural terpenes and antituberculous agents against reference and clinical *Mycobacterium tuberculosis* strains. Molecules.

[30] Silva A.C.R., Lopes P.M., Azevedo M.M.B., Costa D.C.M., Alviano C.S., Alviano D.S. (2012). Biological activities of *α*-Pinene and *β*-Pinene enantiomers. Molecules.

[31] Burt S. (2004). Essential oils: Their antibacterial properties and potential applications in foods. Int. J. Food Microbiol.

[32] Pereira N.L.F., Aquino P.E.A., Júnior J.G.A.S., Cristo J.S.M.A.V., Moura F.F., Ferreira N.M.N., Silva M.K.N., Nascimento E.M., Correia F.M.A., Cunha F.A.B. (2017). Antibacterial activity and antibiotic modulating potential of the essential oil obtained from *Eugenia jambolana* in association with led lights. J. Photochem. Photobiol. B.

[33] Matias E.F.F., Bezerra V.B., Barros R.O., Lucena A.L.V.M., Leite V.D., Feitosa J.H.F., Silva M.K.N. (2017). Avaliação da atividade antibacteriana e moduladora do óleo essencial de *Cordia verbenacea* DC. associado às luzes de LED. Rev. Interfaces.

[34] Caffarel-Salvador E., Kearney M.C., Mairs R., Gallo L., Stewart S.A., Brady A.J., Donnelly R.F. (2015). Methylene blue-loaded dissolving microneedles: Potential use in photodynamic antimicrobial chemotherapy of infected wounds. Pharmaceutics.

[35] Bueno J., Cristofolinli G.M.A.F. (2014). Led terapia na faixa do vermelho ao infravermelho: Uma nova abordagem sob a visão quântica para a saúde. Rev. Saúde Quântica.

[36] Brito S.V., Ferreira F.S., Siqueira-Júnior J.P., Costa J.G., Almeida W.O., Coutinho H.D. (2012). Phototoxic and modulatory effects of natural products from the skin of *Rhinella jimi* (Stevaux, 2002). Rev. Bras. Farmacogn..

[37] Coutinho H.D.M., Costa J.G.M., Siqueira-Júnior J.P., Lima E.O. (2009). In vitro phototoxic activity of *Eugenia jambolana* L. and *Hyptis martiusii Benth*. J. Photochem Photobiol. B.

[38] Ragàs X., He X., Agut M., Roxo-Rosa M., Gonsalves A.R., Serra A.C., Nonell S. (2013). Singlet oxygen in antimicrobial photodynamic therapy: Photosensitizer-dependent production and decay in *E. coli*. Molecules.

[39] Figueiredo F.G., Ferreira E.O., Lucena B.F.F., Torres C.M.G., Lucetti D.L., Lucetti E.C.P., Silva J.M.F.L., Santos F.A.V., Medeiros C.R., Oliveira G.M.M. (2013). Modulation of the antibiotic activity by extracts from *Amburana cearensis* A. C. Smith and *Anadenanthera macrocarpa* (Benth.) Brenan. Biomed. Res. Int..

[40] Wasicky R. (1963). Uma modificação do aparelho de Clevenger para extração de óleos essenciais. Revista da Faculdade Farmácia e Bioquímica.

[41] Adams R.P. (2007). Identification of essential oil components by gas chromatography/mass spectroscopy. Allured Publ. Corp..

[42] NCCLS (2003). Methods for Dilution Antimicrobial Susceptibility Tests for Bacteria that Grow Aerobically; Approved Standard.

[43] Coutinho H.D.M., Costa J.G.M., Siqueira-Júnior J.P., Lima E.O. (2008). In Vitro anti-staphylococcal activity of *Hyptis martiusii* Benth against methicillin-resistant *Staphylococcus aureus*: MRSA strains. Rev. Bras. Farmacogn..

